# Intravascular Papillary Endothelial Hyperplasia Associated with Venous Pool Arising in the Lower Lip: A Case Report

**DOI:** 10.1155/2009/940686

**Published:** 2010-01-05

**Authors:** Hisanobu Yonezawa, Akimitsu Hiraki, Ken-ichi Iyama, Masanori Shinohara

**Affiliations:** ^1^Department of Oral and Maxillofacial Surgery, Sensory and Motor Organ Sciences, Graduate School of Medicine and Pharmaceutical Sciences, Kumamoto University, Kumamoto 860-8556, Japan; ^2^Department of Surgical Pathology, Kumamoto University School of Medicine, Kumamoto 860-8556, Japan

## Abstract

Intravascular papillary endothelial hyperplasia is a benign nonneoplastic vascular lesion that consists of endothelial cells with abundant vascular tissue with papillary proliferation. An adult female had a painless growing dark red nodule on the left side of the lower lip and often touched and gnawed at it for more than 4 years. The lesion was a tender, smooth mass approximately 1 cm in diameter without discoloration reaction. Magnetic resonance imaging of the lesion showed specific findings. She was diagnosed clinically as having mimicked hemangioma, and the lesion was totally excised under local anesthesia. Histopathological examination revealed that papillary proliferated endothelial cells with venous pool, and the lesion was diagnosed as intravascular papillary endothelial hyperplasia associated with venous pool. There has been no recurrence for more than 1 year. Despite the benign nature of this lesion, it could have been mistaken for a malignant tumor because of its clinical course and radiologic findings.

## 1. Introduction

Intravascular papillary endothelial hyperplasia (IPEH) is a benign nonneoplastic and vascular lesion. The lesion was first described by Masson [1] as a *Hemangioendothelioma vegetant intravasculaire* in 1923. IPEH was first named by Clearkin and Enzinger [2] in 1976 as a benign tumor arising in the oral region [3] or other regions of the body [1, 2, 4], and it must be distinguished from other lesions such as simple hemangioma, angiosarcoma [5], malignant endovascular papilloma, and endothelioma [6]. The lesion is also described as intravascular angiomatosis, intravascular endothelial proliferation [7], Masson's intravascular hemangioendothelioma [8], or Masson's pseudoangiosarcoma [9]. Histologically, IPEH consists of endothelial cells with abundant vascular tissue with papillary proliferation. IPEH is rarely found in the oral region [10] and comprises approximately 2% of vascular tumors and subcutaneous lesions [11]. This lesion is considered to result from trauma or stimulation [12], and asymptomatic, grows slowly by discontinuous stimulation such as gnawing, clenching, or suction.

In this report, an adult female was diagnosed as having mimicked hemangioma arising in the lower lip by clinical finding and magnetic resonance imaging, and the lesion was excised completely. The lesion was examined by histopathological and immunohistochemical methods.

## 2. Report of a Case

**Figure 1:**
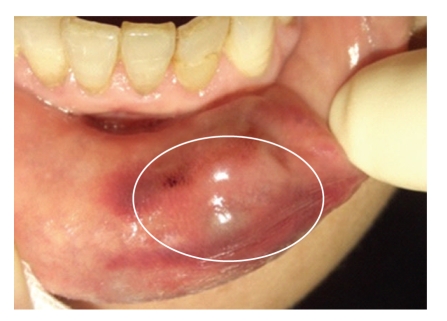
A dark-red-colored tumor, measuring approximately 1 cm in diameter, that was tender and smooth, with a clear boundary being seen in the left side of the lower lip (inside white circle).

A 62-year-old female was referred for a painless, asymptomatic, nonpulsatile slow growing dark red nodule in the lower lip. For more than 4 years the patient was aware of the lesion and she had frequently touched and gnawed around it. There was no contribution of her general medical history, and the head and neck examination revealed no evidence of adenopathy, paresthesia, or motor nerve deficiency. Extraoral findings revealed that the appearance of her skin was normal and symmetrical. Clinical intraoral examination of the left side of the lower lip showed a tender, smooth mass measuring approximately 1 cm in diameter without discoloration reaction (Figure 1). The boundary of the lesion was clear.

**Figure 2:**
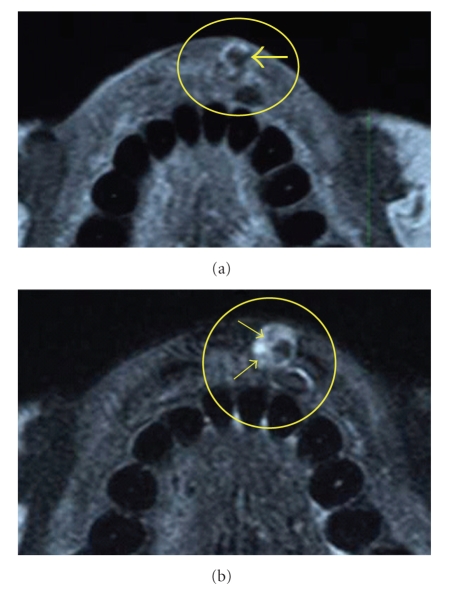
(a) Axial T1-weighed MR image of the lesion (inside yellow circle). This image shows that the margin of the lesion showed an enhanced line with no signal area in the center of the lesion (arrow). TR was 542 (milliseconds) and TE was 9 (milliseconds). The sequence of MR image was FSE, and NEX was 512 × 512. (b) Axial T2-weighed MR image of the lesion (inside yellow circle). This image shows a no signal intensity area in the center of the lesion and also a no signal intensity area beside two low-signal intensity dots (arrows). TR and TE parameters of T2 weighed MR image were 3000 (m sec) and 80 (m sec). The sequence of MR image was FSE, and NEX was 512 × 512.

The lesion mimicked a hemangioma and was examined by magnetic resonance imaging (MR image) and it was not examined by sonography. In an axial T1 weighed MR image, the marginal edge of the lesion showed an enhanced line with the center of the lesion showing zero signal intensity (Figure 2(a)). In an axial T2-weighed MR image, the side edge of the lesion showed a high-signal intensity area and the inside of the lesion also showed a no signal-intensity area next to two low-signal-intensity dots (Figure 2(b)). The MR image mimicked a hemangioma.

The lesion was totally excised under local anesthesia. Surgical procedure revealed that the mass was denuded carefully and removed after the incision around the mass. And the finding revealed that the mass was not clearly continued to the surrounding tissue, there was extremely little bleeding around the mass. The continuity of the mass to the surrounding tissue was not clear. The mass was excised with ease, and minimal blood was lost during the surgery. No recurrence was observed more than 1 year postoperatively. 

**Figure 3:**
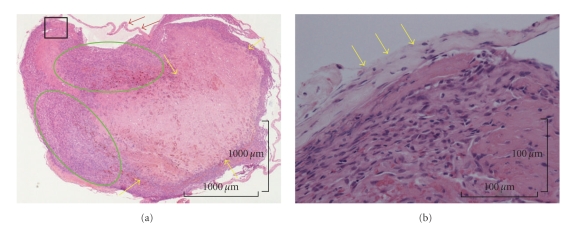
(a) A thrombus (inside green circles) was observed in the expanded blood vessels (blue arrows) with venous pool (yellow arrows) using hematoxylin-eosin staining (Original magnification, ×3.2; Bar, 1000 micro meter). (b) The figure shows the area inside the black square of Figure 2(a). Proliferated endothelial papillary cells were observed. Expanded elastic fibers (yellow arrows) are present on the blood vessel surface using hematoxylin-eosin staining (Original magnification, ×16; Bar, 100 micrometer).

**Figure 4:**
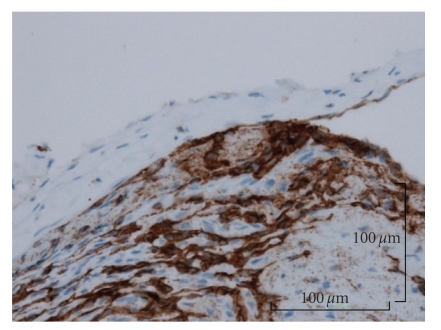
The endothelial cells of this lesion were reactive on immunohistochemical staining for CD31, and CD31 was positive in the endothelial cells (Original magnification, ×16; Bar, 100 micrometer).

**Figure 5:**
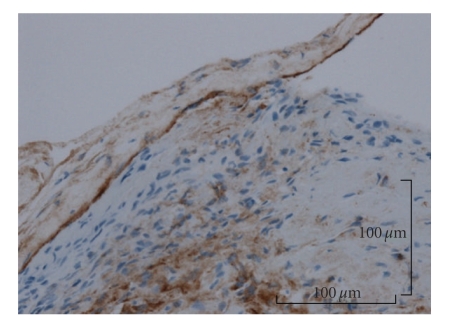
The endothelial cells of this lesion were reactive on immunohistochemical staining for Factor VIII related antigen, and Factor VIII related antigen was positive in the endothelial cells (Original magnification, ×16; Bar, 100 micrometer).

**Figure 6:**
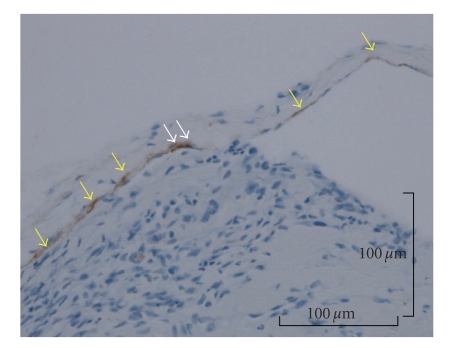
The endothelial cells of this lesion were reactive on immunohistochemical staining for alpha-SMA, and alpha-SMA was positive in the single layer of the blood vessel walls (yellow arrows). Thin, expanded smooth muscle cells in the vessel wall (white arrows) were also observed (Original magnification, ×16; Bar, 100 micrometer).

 According to the histological examination, a thrombus was observed in an expanded blood vessel. In addition, it was observed that the endothelial cells showed papillary proliferation (Figures 3(a) and 3(b)). Expanded elastic fibers on the blood vessel were also observed in Victoria blue staining. Histological examination of the lesion diagnosed it as intravascular papillary endothelial hyperplasia associated with venous pool. And a proliferative process was entirely confined to the vascular space and the papillae was covered by no more than two endothelial cell layers, and mitotic figures and tissue necrosis were absent. The immunohistochemical examination revealed that staining for cluster of differentiation 31 (CD31) (Figure 4) and Factor VIII-related antigen (Figure 5) were positive in the endothelial cells, and staining for alpha-smooth muscle actin (alpha-SMA) was positive in the single layer of the blood vessel walls. Thin, expanded smooth muscle cells in the vessel wall were also observed (Figure 6).

## 3. Discussion

Intravascular papillary endothelial hyperplasia is a benign, non-neoplastic, and vascular lesion [13]. IPEH is considered a posttraumatic phenomenon [12, 14]. In this case, the lesion occurred as a result of stimulation for a long time, with the lesion growing slowly with vascular proliferation arising in the lower lip. IPEH in the oral region is most frequently located in the lower lip (40 of 91 cases) because of the stimulation from biting, touching, and trauma. Other typical locations are the tongue (19 of 91 cases), buccal mucosa (13 of 91 cases), upper lip (12 of 91 cases), mandibular vestibule (12 of 91 cases), and the angle of mouth [10]. Matsuzaka et al. [15] also reported that typical locations of IPEH are the lower lip (47.4%), tongue (15.8%), upper lip (15.8%), buccal mucosa (12.3%), and mandibular vestibule (8.8%). Cases in adult patients aged 26 to 60 years are also more frequent [10]. Histologically, this lesion is characterized by papillary hyperplasia and proliferating endothelium. The histological findings are the deciding factor for the diagnosis of IPEH. 

However, to differentiate IPEH from malignant tumors, such as angiosarcoma [16], malignant endovascular papilloma, and endothelioma, appropriate diagnosis is needed before operation planning [12]. IPEH is distinguished from hrmangioma or other neurogenic tumors, and IPEH is not found in discoloration reaction and any neurogenic history, such as motor nerve deficiency. This lesion, IPEH, is also distinguished from vascular anomaly or primary vascular branch as calibre persistent labial artery (CPLA) [17]. CPLA is similar to IPEH in terms of clinical findings. But CPLA is a pulsatile lesion and vascular anomaly, and IPEH consists of proliferating vascular endothelium; so it is not a pulsatile lesion. Thus, Doppler ultrasonography is a valuable method for diagnosis and eliminates the need for surgical resection. In this case, it was need to perform sonography before surgical resection. 

According to the examination of MR images of hemangioma arising from soft tissues, Teo et al. described that they reviewed MR imaging studies of 22 patients with soft-tissue hemangiomas and 22 patients with malignant soft-tissue masses [18]. They reported that all combinations of lobulation, septation, and central low-signal-intensity dots were specific for hemangioma in T2 images with gadolinium enhancement, and hemangioma is distinguished from malignant sarcoma by these findings. An axial T2-weighed MR image of the lesion revealed that the side edge of the lesion had a high-signal intensity area and the central part of the lesion showed a no signal-intensity area in addition to two low-signal-intensity dots with lobulation and septation. Thus, this lesion was distinguished from hemangioma as it showed an area of zero signal intensity. Furthermore, this lesion was also distinguished from angiosarcoma because the dots were not located centrally in an area of lobulation. The findings by MR imaging and clinical finding such as nondiscoloration reaction of the lesion revealed that it was atypical or mimicked hemangioma or another non-neoplastic lesion. In the MR images, Figure 2 shows a high-signal intensity area and a no signal intensity area beside two low-signal-intensity dots. A high-signal intensity area corresponds to the endothelial cells with proliferation, a no signal intensity area corresponds to venous pool and two low-signal-intensity dots correspond to a venous pool with thrombi in Figure 3. 

In the immunohistochemical examination of IPEH, Albrecht et al. described that IPEH is closely related to organizing thrombi using antibodies to Factor VIII-related antigen, ferritin, and vimentin, Bodner and Dayan [3, 4]. In addition, Suster and Wongalso described that the staining for CD34 is positive in the normal blood vessel walls with high sensitivity and is also positive in the well-differentiated, mature blood vessel specifically [19]. And Soares et al. described that the staining for CD34 is positive in some of endothelial cells, but the staining for CD105 is negative, and Collagen type IV and laminin are positive in the basement membrane of the vessel wall and SMA is positive in some cells surrounding the vessel wall. The staining for vimentin is strong positive stroma. Collagen type I is present in the stroma including the hyalinized areas [16]. In this case, we examined this lesion using staining for CD31, Factor VIII-related antigen, and alpha-SMA. The examinations for CD31 and Factor VIII-related antigen were positive in the endothelial cells (Figures 4 and 5), and because the staining for alpha-SMA was also positive, thin, expanded smooth muscle cells in the vessel wall were observed. The area of positive thin area of alpha-SMA revealed that the normal vessel wall was pulled thin by proliferating endothelial cells and thrombi (Figure 6). The examination of CD31 in the endothelial cells of this lesion showed that the mass was of blood vessel origin and the endothelial cells were proliferating to the inside vessel wall (Figure 4). Including examination for Factor VIII-related antigen and alpha-SMA, we concluded that the tumor was of blood vessel origin with thrombus and a thin layer of smooth muscle cells around the endothelial cells. Furthermore, Tosios et al. also described that the presence of Factor VIII-related antigen in the final stage of organization was characteristic of IPEH [11] and the presence in this case is proof of IPEH. 

In conclusion, we diagnosed this lesion as IPEH with venous pool because the histopathological and immunohistochemical findings revealed vigorous proliferation and papillary structures of endothelial cells associated with thrombi. In this study, we examined this lesion by MR imaging and the diagnosis was based on histological and immunohistochemical findings.
